# Genome Sequencing for Genetics Diagnosis of Patients With Intellectual Disability: The DEFIDIAG Study

**DOI:** 10.3389/fgene.2021.766964

**Published:** 2022-02-01

**Authors:** Christine Binquet, Catherine Lejeune, Laurence Faivre, Marion Bouctot, Marie-Laure Asensio, Alban Simon, Jean-François Deleuze, Anne Boland, Francis Guillemin, Valérie Seror, Christelle Delmas, Hélène Espérou, Yannis Duffourd, Stanislas Lyonnet, Sylvie Odent, Delphine Heron, Damien Sanlaville, Thierry Frebourg, Bénédicte Gerard, Hélène Dollfus

**Affiliations:** ^1^ Inserm, CHU Dijon-Bourgogne, CIC1432-Epidémiologie Clinique, Dijon, France; ^2^ CHU Dijon-Bourgogne, Fédération Hospitalo-Universitaire Médecine Translationnelle et Anomalies Du Développement (TRANSLAD), Dijon, France; ^3^ Inserm, Université de Bourgogne-Franche-Comté, UMR 1231, EPICAD, Dijon, France; ^4^ Inserm, Université Bourgogne-Franche-Comté, UMR1231, équipe GAD, Dijon, France; ^5^ Service de Génétique Clinique, Centre de Référence Anomalies Du Développement CLAD Est, CHU de Dijon, ERN ITHACA, Dijon, France; ^6^ Service de Génétique Médicale, Institut de Génétique Médicale D’Alsace, Hôpitaux Universitaires de Strasbourg, Strasbourg, France; ^7^ Université Paris-Saclay, CEA, Centre National de Recherche en Génomique Humaine (CNRGH), Evry, France; ^8^ CIC1433-Epidémiologie Clinique, Inserm, Centre Hospitalier Régional et Universitaire de Nancy, Université de Lorraine, Nancy, France; ^9^ Aix Marseille Université, IRD, APHM, SSA, VITROME, IHU-Méditerranée Infection, Marseille, France; ^10^ Inserm, Pôle de Recherche Clinique, Paris, France; ^11^ Université de Paris, INSERM, IHU Imagine–Institut des maladies génétiques, Paris, France; ^12^ Fédération de Génétique et Médecine Génomique, GHU APHP.centre-Université de Paris, Hôpital Necker-Enfants Malades, Paris, France; ^13^ Service de Génétique Clinique, Centre de Référence Anomalies Du Développement CLAD- Ouest, Université de Rennes, CNRS, IGDR (Institut de Génétique et Développement de Rennes), ERN ITHACA, Rennes, France; ^14^ Unité Fonctionnelle de Génétique Médicale et Centre de Référence « Déficiences Intellectuelles de Causes Rares », APHP Sorbonne Université, Groupe Hospitalier Pitié-Salpêtrière et Hôpital Trousseau, Paris, France; ^15^ Hospices Civils de Lyon, GHE, Service de Génétique, Université Claude Bernard Lyon 1, Lyon, France; ^16^ CHU de Rouen, Service de Génétique, Rouen, France; ^17^ Inserm, Université de Normandie, UMR1245, Centre de Génomique et de Médecine Personnalisée, Rouen, France; ^18^ Laboratoires de Diagnostic Génétique, Institut de Génétique Médicale D’Alsace, Hôpitaux Universitaires de Strasbourg, Strasbourg, France; ^19^ Inserm UMRS_1112, Institut de Génétique Médicale D’Alsace, Université de Strasbourg, France et CHRU, Strasbourg, France

**Keywords:** genome sequencing, intellectual disability, cost-effectiveness, minimal reference strategy, diagnostic odyssey

## Abstract

**Introduction:** Intellectual Disability (ID) is the most common cause of referral to pediatric genetic centers, as it affects around 1–3% of the general population and is characterized by a wide genetic heterogeneity. The Genome Sequencing (GS) approach is expected to achieve a higher diagnostic yield than exome sequencing given its wider and more homogenous coverage, and, since theoretically, it can more accurately detect variations in regions traditionally not well captured and identify structural variants, or intergenic/deep intronic putatively pathological events. The decreasing cost of sequencing, the progress in data-management and bioinformatics, prompted us to assess GS efficiency as the first line procedure to identify the molecular diagnosis in patients without obvious ID etiology. This work is being carried out in the framework of the national French initiative for genomic medicine (Plan France Médecine Génomique 2025).

**Methods and Analysis:** This multidisciplinary, prospective diagnostic study will compare the diagnostic yield of GS trio analysis (index case, father, mother) with the French core minimal reference strategy (Fragile-X testing, chromosomal microarray analysis and Gene Panel Strategy of 44 selected ID genes). Both strategies are applied in a blinded fashion, in parallel, in the same population of 1275 ID index cases with no obvious diagnosis (50% not previously investigated). Among them, a subgroup of 196 patients are randomized to undergo GS proband analysis in addition to GS trio analysis plus the French core minimal reference strategy, in order to compare their efficiency. The study also aims to identify the most appropriate strategy according to the clinical presentation of the patients, to evaluate the impact of deployment of GS on the families’ diagnostic odyssey and the modification of their care, and to identify the advantages/difficulties for the patients and their families.

**Ethics Statement:** The protocol was approved by the Ethics Committee Sud Méditerranée I and the French data privacy commission (CNIL, authorization 919361).

**Trial Registration:** ClinicalTrials.gov identifier NCT04154891 (07/11/2019).

## Introduction

The advent of Next Generation Sequencing (NGS) technologies has revolutionized our approach to diagnosis and research in the field of rare diseases at an international level, prompting rapid efforts to deploy these technologies in many countries, and by the European Commission (“1 + Million Genomes Initiative”, launched in 2018). In the same spirit, the 2016 launch of the French plan for genomic medicine (AVIESAN, 2016) was designed to serve a range of medical disciplines, including cancer and rare diseases. The France Genomic Medicine 2025 plan cited rare diseases as one of the key areas at the forefront of NGS implementation, to improve patient care, shorten their diagnostic odyssey and boost research. Indeed, genetic diagnosis is the first step towards appropriate care, follow-up and genetic counseling. Moreover, enhancing our understanding of pathogenesis could help to elaborate more specific therapies. The general goal of French Genomic Medicine plan is to implement the use of Genome Sequencing (GS) in France within the next 10 years, by creating national sequencing platforms (2 of which are now fully operational), and as a kick off, to implement four pilot studies, one of which is dedicated to rare diseases, namely the DEFIDIAG pilot study.

This DEFIDIAG pilot study focuses on intellectual disability (ID), as one of the most challenging models of rare disease. ID affects around 1–3% of the general population, with around 15 per 1,000 persons having mild ID and around 3 per 1,000 having severe ID. It is the most common cause of referral to pediatric genetic centers. ID results from abnormal brain development due to numerous possible cellular processes, including neuron proliferation and differentiation, neuron or astrocyte metabolism and maintenance, neurotransmitter synthesis, receptor or signal transduction, transcriptional and translational control. It is also well recognized that ID may result from non-genetic causes (neonatal anoxia, toxic effects, deprivation … ) or from genetic causes. Gene and chromosomal variations involved in human ID are numerous and include recurrent chromosomal events from complete chromosomal duplication, segmental chromosomal duplication or deletion, or small genic variations altering the function one among more than 1,500 genes such as single nucleotide variants (SNV), insertions or deletions (indel), unbalanced (CNV) or balanced structural variants (SV) in coding or non-coding regions, or even rarer events such as repeat expansions, uniparental disomy, mobile element insertion etc., which are reported in the OMIM registry. Approximately 15% of ID is attributable to cytogenetically clear-cut abnormalities, with at least two-thirds of these cases accounted for by trisomy 21 (Ellison et al., 2013) and up to 40% to one variation in one of the 1500 ID gene when exome sequencing is performed (Han and Lee, 2020).

Before next generation sequencing methods, genetic testing was limited to traditional karyotype and fragile X analysis, sometimes with gene specific Sanger analysis, when a specific syndromic ID was suspected. In the past 10 years, microarray analysis (CMA) has been widely used for the genetic diagnosis of ID. NGS tests recently emerged, in the last 5 years, with gene panel approaches and Whole Exome Sequencing (ES) following CMA and fragile X syndrome screening. Today, the question arises about the input of whole genome sequencing as a first approach in such patients, as this may profoundly modify the testing process by replacing CMA and other sequencing approaches (specific genes, panel, ES) and in addition, may detect new molecular mechanisms not detectable by CMA and ES.

Nowadays, most patients with no clinical diagnosis in France still undergo basic investigation (Fragile X and CMA) with a diagnostic yield of less than 20%. This basic exploration is usually followed by additional analysis using gene panel approaches containing at the very least a minimal core of 44 genes, namely the 44GPS minimal list recommended by the French national association of molecular genetics practitioners (Association Nationale des Praticiens de Génétique Moléculaire, ANPGM). The panel sequencing in the proband gives an additional diagnostic yield of 10–12% (using the 44 gene panel), and up to 40% (Han and Lee, 2020) in case of ES. ES as the first line test for the diagnosis of rare genetic diseases has recently been shown in some countries to be cost-effective, tripling the diagnostic rate at one third of the cost, in children with suspected monogenic disorders (Stark et al., 2017; Schofield et al., 2019). There is also emerging evidence of the efficiency of GS over standard testing (Alam and Schofield, 2018). However, the heterogeneity in clinical presentation, sample sizes and health economic evaluation standards makes it difficult to generalize the first published results (Schwarze et al., 2018) to other settings. A recent study in Ontario, Canada reported that GS might have a higher diagnostic yield than standard genetic testing, and could be a cost-effective strategy when used after standard testing or when used earlier in the diagnostic pathway (Ontario Health (Quality), 2020). However, these results need to be confirmed in the French setting since marked differences exist between countries in the technologies used, the costs, and the organization of clinical, biological and bioinformatics pathways.

Against this background, the DEFIDIAG study aims to evaluate the diagnostic performance and cost-effectiveness of GS as a systematic and unique molecular investigation for French patients with ID of unknown etiology, compared to the standard minimal protocol defined by the ANPGM, under conditions close to routine. Indeed, GS provides an opportunity to analyze a wider panel of molecular events, such as: 1) SNV and insertion/deletion (indel) in coding regions, even in CG rich regions, 5′ and 3′ UTR, promoter or intronic regions; 2) unbalanced chromosomal anomalies (CNV), with greater accuracy due to homogeneous coverage; 3) balanced structural variants, such as inversion and translocation, and lastly, 4) mechanisms observed very infrequently, such as uniparental disomy for imprinted chromosomal regions or insertion of mobile elements. Genome analysis was chosen because potential splice site mutations in deeper intronic regions are not enriched by exome analysis; CNV in exome enrichment strategies are still not 100% sensitive, specific and reliable; partial inversions or translocations affecting coding regions are not found by ES, and promoter or regulatory regions are not analyzed by ES.

This study also includes impact studies, aiming to assess the perceived impact for patients and their parents, for whom this technique may herald the end of their diagnostic odyssey. To this end, the DEFIDIAG study involves 15 medical genetics departments with strong clinical expertise in ID patients, as well as six diagnostic laboratories with a proven track record of competence in ID gene exploration, and the national sequencing platform of the National Center of Human Genomics Research (CNRGH), recognized for the high quality of its genomic sequence production and bioinformatics processes.

Specifically, the primary objective of this study is to compare the percentage of causal genetic diagnosis identified by GS performed on a trio (the patient and both parents) (GST), to the use of the current French reference minimal strategy (Fragile X + CMA + 44GPS) in ID patients attending a first genetics consultation. Secondary objectives focus on the diagnostic yield, and include the following:

• To compare the percentage of ID causal diagnosis identified by GST, to GS in the proband only (i.e., genome in solo, GSS) in a subgroup of randomized patients attending for a first genetic investigation. This evaluation is useful because in genetic counseling, both parents are not always available.
• To describe the estimated additional diagnostic yield that would be obtained at each step in sequential analysis of the GS data: for the minimal 44GPS gene panel, the list of genes known to be associated with disease from OMIM (referred to simply as OMIM), and ES, in patients attending for a first investigation (never-explored patients) and in patients who have already undergone investigations (previously-explored patients).
• To compare the percentage of causal diagnosis of ID identified by GST to that obtained with the French reference minimal strategy in various subgroups (defined according to age, severity of ID, presence of major non-cerebral manifestations or epilepsy), for patients with ID attending for a first genetic investigation (never-explored patients).
• To compare the percentage of causal structural changes (CNV, balanced structural variants) identified by GST versus CMA.
• The DEFIDAG study also aims to compare the reference strategy, GST and GSs, in terms of costs and effectiveness for the causal diagnosis of ID in patients with ID of unknown etiology, attending for a first genetic investigation.
• Finally, three impact studies are planned. The first will estimate the costs associated with searching for a molecular diagnosis that could potentially be avoided by performing GS as the first line approach. The second will evaluate the impact on the frequency and type of medical, medico-social and psychological care in the year following the release of GS results, compared to care during the year prior to inclusion, in particular in patients who were already engaged in a diagnostic process prior to being invited to participate in the DEFIDIAG study. The third impact study will be performed in two centers, and will use a qualitative approach, namely interviews with a sample of parents, to explore: 1) the burden experienced by the parents, 2) emotional adjustment of the patient and the parents to the results of genetic tests, and 3) the patient and parents’ perception of the future.

## Methods and Analyses

### Study Setting

The DEFIDIAG study is a prospective multicenter diagnostic study comparing two main strategies (namely GST versus the minimal reference strategy) applied in a blinded fashion to consecutive patients with no obvious clinical diagnosis, referred to medical genetic departments. Each patient included serves as their own control, and will undergo both strategies being compared. In parallel, the percentage of ID causal diagnosis identified by GSS, as compared to GST, will be evaluated in a randomized subgroup of patients attending a first genetic investigation.

**FIGURE 1:**
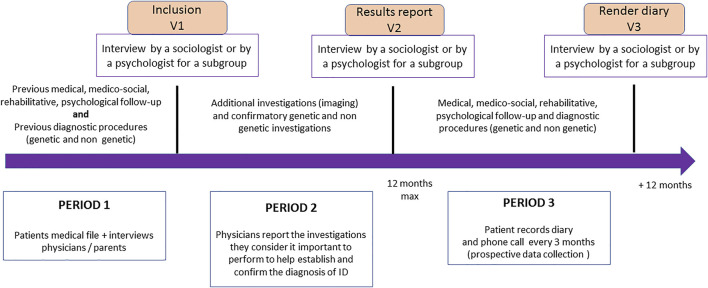
Impact study procedures and schedule (DEFIDIAG study). The figure represents the key timepoints for patient visits in the inclusion centers (inclusion in the study, delivery of results, 12-months post-delivery visit), which also correspond to the time of the interviews between either a sociology or psychology researcher and the parents/patients who agreed to participate in the qualitative impact study. The figure also shows the three periods considered for cost estimation (period 1: before inclusion; period 2: waiting for results; period 3: during the 12 months following the results) and, for each of these periods, the different elements collected from the care teams and families in order to have the examinations carried out (for the three periods) and those envisaged with each of the strategies to confirm the diagnosis (period 2).

In addition to the prospective diagnostic study, we will also perform quantitative impact studies to collect data concerning management of patients before inclusion, during the genetic analyses process, and after the results are made known (Figure 1); as well as a qualitative substudy using interviews (one interview after inclusion, a second interview after the results are made known to the parents/patients, and a third and final interview 12 months after the results are made known) in a sample of parents. The target population for each objective is described in Table 1.

**TABLE 1:** Correspondence between objectives and target population (DEFIDIAG study).

Eligible index cases (*n* = 1,275)	
**Index cases with undiagnosed ID coming for the first time for a genetic investigation (*n* = 637)**	**Index cases with undiagnosed ID already investigated (*n* = 637)**
Primary objective: Compare the percentage of genetic causal diagnosis identified in ID patients by performing trio GS analysis vs. the use of the current French reference minimal strategy	
Secondary objective: diagnostic yields	
•Compare the percentage of ID causal diagnosis identified by GST vs. the reference strategy in different subgroups (defined according to age, or clinical manifestations)	
•Compare the percentage of causal structural changes identified by GST vs. chromosomal microarray analysis	
•Compare the percentage of ID causal diagnosis identified by GST vs. GSS in a subgroup of randomized patients (*n* = 196)	
Secondary objective: Describe the estimated additional diagnostic yield that would be obtained at different steps of sequential analysis, for the 44 gene panels, OMIM and exome analysis	
Secondary objective: Assessing the efficiency of the three strategies (the reference minimal strategy; GSs; GS_T_)	
	Secondary objective: Estimate the cost of the diagnostic odyssey that could be potentially avoided by first-line genomic analysis
Secondary objective: Estimate the frequency and nature of changes in medical follow-up of the patients, but also in medico-social, rehabilitation, and psychological follow-up in the first year after the reporting of GS analyses compared to the period before the inclusion	
Secondary objective: Evaluate the burden experienced by the parents, 2) the parents’ emotional adjustment to the primary genetic tests results, and 3) the parents’ perception of the future	

GS_T_: genome sequencing—trio analysis; GS_S_: genome sequencing—proband only.

### Strategies Compared

**FIGURE 2:**
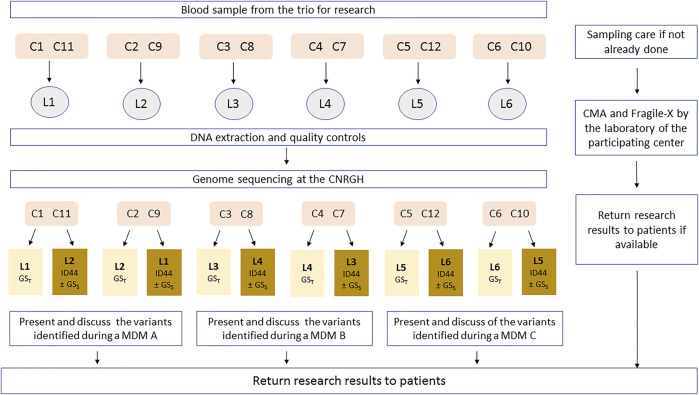
Sample flow description (DEFIDIAG study). C: Clinical centers; L: Reference laboratory (highlighted in light yellow) and Mirror Laboratory (highlighted in dark yellow); MDM: MultiDisciplinary meeting; CNRGH: Centre National de Recherche en Génomique Humaine (National Centre for Human Genomic Research). Each clinical center (C, numbered 1–12) is affiliated to one reference laboratory (numbered 1–6) in charge of the analysis of trio-genome sequencing—GST; each laboratory (L) is affiliated to 2 clinical recruitment centers as a reference laboratory (for example: L1 will be the reference laboratory for patients from C1 and C2) and will work in pairs with another laboratory (mirror laboratories), in charge of ID44 and of the analysis of solo-genome sequencing (GSS) for patients randomized in the appropriate sub-group. This mirror laboratory is itself affiliated with two other recruitment centers (for example: L1 will be the mirror laboratory for patients from C3 and C4). The 4 clinical centers organize the multidisciplinary meeting (MDM) together with their 2 official laboratories.

In order to avoid producing redundant sequences, for each patient included, one unique set of genomic data sequences will be produced by a unique sequencing platform (CNRGH, Evry, France). Analyses are then performed blindly by two independent mirror laboratories: one laboratory will analyze the genome with the parental inheritance information (GST), while the second will analyze only the 44 genes of the reference minimal strategy (44GPS), without parental inheritance information, as well as the proband genome (GSS) in a randomized selection of patients (Figure 2; Supplementary Table S1).

The results of the 44GPS will be withdrawn from the GS data for all patients, using a specific bioinformatics filtering procedure that mimics the results obtained using targeted capture analysis (exons ± 20 intronic bases, SNV and CNV analysis). Fragile-X and CMA (included in the reference strategy) follow the routine care circuit, which is mainly independent of the GS circuit.

GST analysis is performed following a harmonized, consensus protocol adopted by the six clinical laboratories involved in this study, and using a common web interface (Polyweb.fr, developed in-house at the Genetic diseases IMAGINE Institute, Paris, France). All genomic variations are called by standard callers (GATK and BWA) and are subsequently ranked using several factors to facilitate analysis (protein impact, inheritance model, ID known gene and control data bases, splice prediction effect … ) in the Polyweb interface. For the SNV/small indel, the scheme contains a first minimal filtration step (for SNV/indel, elimination of the variations with over 1,000 hits in GnomAD, or 5 hits at homozygous state). All variations with a predicted protein impact are ranked at the top of the list and will be studied even when a first deleterious causative variation is detected. This broad approach ensures that co-occurring mutations in different genes will be identified [multi allelism is expected in about 5% of the cases (Yang et al., 2014)]. Additional bioinformatics modules such as repeat tracking, promoter and evolutionary conserved regions analysis, and mobile element insertion detection will be implemented and applied onto negative genome results during the ongoing protocol. Specific protocols for the proband GS data and 44GPS analysis are also developed and shared between the six laboratories. To limit time dedicated to GS proband analysis, SNV/indel analysis only focuses on exonic ± 20 bases of the known ID genes (SysID list) and OMIM disease-associated genes.

When the analysis is completed, variants of interest (highly suspected to be pathogenic) are recorded in a dedicated and secured electronic Case Report Form (e-CRF). Variant class (pathogenic as class 5 variant, probably pathogenic as class 4 variant or of unknown significance but highly suspected to be pathogenic as class 3 + variant) is validated during a Multi-Disciplinary Meeting (MDM) before being communicated to the patient.

The results of the microarray analysis and Fragile X analysis will be communicated to families and collected in the dedicated e-CRF by the recruiting genetic team as soon as the results are obtained from the diagnostic laboratories. The laboratories in charge of the GS analyses will remain blinded to these results. The GS results will be available approximately 6–9 months after inclusion in the study. In the event of an emergency (such as pregnancy), the blinding can be lifted and the results delivered to families as soon as they become available.

### Study Endpoint

#### Primary Outcome

The primary study endpoint is the identification of a causal diagnosis of ID, defined as the identification of one or more class 4 or 5 variant(s) that explain the symptoms presented by the patient, and validated during a specific MDM.

#### Secondary Outcomes

• Efficacy in terms of diagnostic yield: identification of causal structural changes
• Efficiency: estimation of the incremental cost-effectiveness ratio, expressed in terms of cost per additional positive diagnosis.
• Quantitative impact studies
• Cost of the diagnostic odyssey: mean cost related to the iterative search for a diagnosis in the previously investigated population.
• Change in follow-up: criteria will be the change in the number and type of medical, medico-social, rehabilitative, and psychological care induced by the results of the trio genomic analyses.
• Qualitative impact studies: A sociologist and a clinical psychologist will explore the consequences on the family, and on the personal, professional and social life of the parents who take care of a child or an adult with ID, as well as the emotional adjustment and possible information overload for the parents at the various stages of the study (inclusion, results rendering, 12 months after receiving results).

### Population

The DEFIDIAG study will be conducted in 15 clinical genetic centers in France. All patients consulting a geneticist in one of the participating centers will be screened for eligibility. The study will include children or adults with ID of unknown etiology (index cases or probands), whatever the severity (but with proven ID by ad hoc neuropsychological testing in patients in whom ID is clinically questionable), and whatever the associated manifestations. Individuals with an obvious ID syndrome with a well-known molecular diagnosis will not be considered for inclusion. Children aged between 0 and 5 years will only be considered for inclusion in case of severely delayed development in terms of motor skills, language, and/or sociability. Patients and both biological parents are included if they confirm their willingness to comply with all the study procedures, their availability for the duration of the study, and sign the appropriate consent forms.

Non-inclusion criteria include: isolated learning disabilities; no possibility of obtaining a blood sample from both biological parents; any in the patient condition that, in the investigator’s opinion, would jeopardize compliance with the protocol; one or both parents with ID; parent placed under judicial protection (guardianship, curatorship, tutorship).

The strategy based on GST is expected to yield a diagnosis in at least 60% of patients with ID versus 30% with the reference minimal strategy in the population of patients attending for a first genetic investigation (never-explored patients). However, the strategies will be compared in 7 subgroups: 1) 3 defined by age: children <2 years old/2–5 years/>5 years; 2) 4 subgroups of patients defined according the severity of ID, and/or 3) with associated manifestations:1) mild ID associated with another sign, 2) moderate to severe ID, 3) ID with major non-cerebral abnormality, and 4) ID associated with epilepsy The smallest sub-group (subjects with mild ID associated with another significant sign) should represent around 15% of the total population, and the difference between the GST and the reference strategy in this particular subgroup can be assumed to be less than 15%. In addition, it is assumed that fewer than 1% of diagnoses identified with the referral minimal strategy will not be identified by the GST. Finally, we will also compare GST and GSS in a subgroup of randomized patients referred for the first time. For this specific comparison, a difference of 7% is expected, and <0.1% of diagnoses identified by GSS and not by GST. Considering these 9 planned comparisons (8 comparisons between GST and the reference strategy and 1 between GST and GS), the one-sided alpha risk is fixed at 0.00278. Based on these hypotheses, and a power of 80%, among patients seen for a first genetic investigation (50% of the population to be recruited), 41 patients are required for the main comparison, and 91 patients with mild ID + other syndrome are necessary. To recruit this number of patients, we estimate that it will be necessary to screen 607 patients attending for a first referral. A subgroup of 196 patients will be randomized to undergo GSS in addition to GST plus the reference minimal strategy. The sample size for this subgroup will be sufficient to compare GST and GSS in terms of diagnostic yield, as well as in terms of efficiency (a sample size over 150 is usually deemed sufficient). As we intend to include 50% of patients attending for a first genetics evaluation (never-explored patients), and 50% of patients who have already been investigated (previously-explored patients), a total of 1,214 patients are necessary. Considering that approximately 5% of samples will not be analyzable, it will therefore be necessary to include 1,275 index cases plus both their parents, for a total of 3,825 participants.

The qualitative study will be performed in a sub-sample of the overall population, in two centres. In order to maximize the scientific rigor of the study and to alleviate the potential burden resulting from the interviews, whilst fostering discussions between the sociological and psychological sides, we chose to separate the interview procedures into two groups. For the psychological sub-study, 15 interviews with parents will be conducted at each assessment timepoint (T1, T2, T3), giving a total of 45 interviews. For the sociological sub-study, 15 interviews with parents will be conducted at each assessment timepoint (T1, T2, T3), giving a total of 45 interviews. A total of 15 interviews per assessment timepoint was chosen to achieve data saturation, a key concept of qualitative analysis, namely the point beyond which further interviews do not yield any new information (Glaser and Strauss, 1967). As far as possible, the study populations of patients and parents will be stratified into two subgroups, namely never-explored, and previously-explored patients. The subgroups of parents will also be stratified according to the clinical profile of the patients (mild vs moderate or severe ID).

### Study Conduct

#### Inclusion

During a genetics consultation and after verifying the inclusion and non-inclusion criteria, the patient and both parents will be informed about the study by the investigators and invited to participate. If they agree either during the visit or after a period of reflection, they will be invited to sign the consent form. During this visit, the history of the illness, family history, the examinations already carried out and the corresponding results will be collected with the assistance of a Clinical Research Technician (CRT) in the dedicated e-CRF prepared by the DEFIDIAG methodology and management center (INSERM Clinical Investigation Center—Clinical Epidemiology Unit CIC-EC1432), using CleanWEB software (Telemedecine technologies SAS, Boulogne-Billancourt, France). If necessary, the clinical geneticist will prescribe additional neuropsychological tests. A blood sample (5 ml -EDTA tubes) will be obtained from each participant (patients with ID as well as both biological parents). These blood samples will be sent to the reference DEFIDIAG laboratory of the center (Figure 2, Supplementary Table S1) in compliance with regulations for the shipment of diagnostic samples (category B). A diary will be given to the family where they will be asked to note the patients’ use of healthcare and medico-social services until the return of the GS results. This diary makes it possible to collect the medical examinations, biological investigations, rehabilitation and psychological consultations as well as the medico-social follow-up. A follow-up by phone will be performed every 3 months by the CRT in order to guarantee the completeness of the collection.

For parents/patients willing to participate in the qualitative study in the two centers conducting this part of the study, additional data concerning the familial situation, the number of children, the social deprivation level and the existence of informal and/or professional caregivers at home will be collected, as well as the contact details and address of the participants, for the sociologist or psychologist to organize the interview.

#### Genomic Circuit

All blood samples received for the DEFIDIAG study by the reference laboratory are extracted using a method previously validated by the sequencing platform (Centre National de Recherche en Génomique Humaine, CNRGH). This validation step was performed on the same blood sample, and genomic sequences obtained after various DNA extraction methods were compared by the CNRGH in terms of global DNA quality, mean coverage and SNV/CNV detection. In all, five extractions methods were validated (three automatic and two manual). After blood extraction, 3 µg DNA aliquots labelled with an anonymous barcode are sent to the CNRGH *via* a courier at room temperature. Several quality controls are then performed before the sequencing step (fluorimetric DNA quantification, quality measurement using the DNA integrity number, PCR amplification test, and sex control). If DNA of the trio is accepted, 1.1 µg of DNA is fragmented using an optimized CNRGH GS protocol. The GS is optimized in order to reach a mean coverage of 30X for each sample; a minimum of 25X mean coverage is required. Below these specifications, the sequencing is considered as a failure and will not be repeated.

### Variant Calling

Whole genomic sequences are analyzed by two separate SV and SNV/indel pipelines developed and validated by the CNRGH sequencing platform and IMAGINE bioinformatics team. Briefly, the raw data will be produced as compressed FASTQ files generated from the. bcl files by the CNRGH sequencing platform. The sequences are aligned to the human reference genome GRCh37 using the Burrows-Wheeler Aligner BWA software (Li and Durbin, 2010) and made available as BAM files. Aligned sequences are sorted, cleaned and the PCR duplicates are marked using the Sambamba software (Tarasov et al., 2015) in order to eliminate most of the NGS’ well-known biases. A local realignment of the sequences around insertion and deletion sites and the base quality recalibration is performed using GATK (McKenna et al., 2010). After sequence quality control and alignment of the reference genome, the CNRGH performs the variant calling on the entire genome for the Single Nucleotide Variants (SNV), small insertion/deletions (indels) and structural variants (including Copy Number Variant, CNV). SNV and indel calling are performed using the Haplotype Caller from GATK software in “bp resolution” mode to produce gVCF files. Imbalanced SV (CNV) detection > 1 kb is performed using three different softwares: Wisecondor (Raman et al., 2019), Canvas (Roller et al., 2016) and Manta (Chen et al., 2016). Balanced SV (translocation, inversion) detection is done using Manta software. Results are produced in the format of a VCF file to match the common file standard format in NGS analysis. These files are then collected by the IMAGINE Polyweb platform: additional combined TRIO gVCF analysis (genotypeGvcf) and CNV Wisecondor analysis will also be performed.

### Quality Controls

Several quality controls are carried out before biological analysis: genome mean coverage over 25X is required for the trio; sex verification (SRY detection) and trio concordance (<1% of Mendelian error transmission in trio using Plink software) are checked before interpretation.

### Biological Analysis

The .vcf and .bam files are implemented in Polyweb software developed and previously validated by the IMAGINE bioinformatics platform. This software makes it possible to annotate, analyse and visualise all the genomic variations in two different web interfaces Polyviewer (for SNV, small indel, exonic deletion or duplication) and Polycyto (for balanced and unbalanced SV) of all human genes in trio or solo analysis. Moreover, a specific ID44 bioinformatic gene panel will make it possible to study variations from this gene list. Read variations are visualized using IGV software (Robinson et al., 2011).

1) SNV/indel analysis

The polyviewer interface gives access to several annotations, such as patient and trio sequencing data (number of mutated and total reads), data from common free-access databases (GnomAD (Karczewski et al., 2020), Clinvar (Landrum et al., 2020), OMIM (Amberger et al., 2019), GenCode (Frankish et al., 2019) …), licensed database HGMDpro and also internal databases (Déjà Vu), gene or protein predicted impact, splice prediction [SpliceAI (Jaganathan et al., 2019)], and for trio analysis, inheritance status of the variation. Our objective is not to evaluate the value of GS for early detection of ID, but rather, to evaluate the risk/benefit of GS for the etiological diagnosis in already-identified ID. The internal database (Déjà Vu) contained over 20.000 exomes, 50.000 panels and 1,000 genomes for SNV/Indel variations with differentiation between ID and non-ID patients.

The following filtration keys are applied to focus on potentially pathogenic variations: GnomAD allele count<1,000, GnomAD homozygote count< 5, and predicted protein impact onto all gene transcripts (Stop gain, Stop loss, Start loss, frameshift, in frame deletions or insertions, missense, and predicted splice region, Déjà Vu for patients non ID < 1,000 and homozygote count < 5).

Ranking of identified variations is then performed based on internal Polyweb criteria: variation sequence quality, *de novo* status if available, known ID gene or OMIM gene, protein or splicing impact prediction, gene with AR inheritance and homozygous or compound heterozygous variations, male and X linked variation, known pathogenic variations in HGMDpro or ClinVar, frequency in GnomAD.

Those criteria will ensure that all variations are analyzed from all known human genes (OMIM or not) that are predicted to affect proteins.

2) SV analysis

The polycyto interface gives access to several annotations using AnnotSV software (Geoffroy et al., 2018), DGV, OMIM and internal Déjà Vu databases. The internal SV database (Déjà Vu) contained at the beginning of the project 200 Novaseq sequenced genomes from non-ID patients. The number of genomes in Déjà Vu is now up to 2000 (mostly ID patients).

Ranking of identified variations is based on calling quality and inheritance status. For balanced SV (translocation and inversion) a greater weight is given to variations whose break points are found in OMIM genes.

After filtering CNV already detected at least 10 times in the Déjà Vu database, all detected CNV are analyzed using standard criteria [ACMG recommendations (Riggs et al., 2020)]. For Déjà Vu count, two CNV are considered identical if they overlap over 75% of their reciprocal length. For balanced SV (translocation and inversion), break points must have an identical genomic position ± 50bp.

All imbalanced and balanced SV are checked in IGV software by visualizing paired read alignment anomalies (insert size, pair orientation and split read). In addition, for CNV, allele frequency plots ranked according to chromosomal positions are also available.

### Biological Interpretation

Biological interpretation follows standard criteria [ACMG recommendations (Richards et al., 2015; Riggs et al., 2020)]. Briefly, balanced and imbalanced SV and SNV/Indel/Small exonic deletions, or duplications are all performed by two independent biologists. All variations are checked on. bam data and doubtful variations are confirmed using standard molecular analysis before biological analysis (less than 10 mutated reads for SNV, doubtful *de novo* status, doubtful deletion, duplication or translocation/inversion … ).

For CNV (duplication, deletion), current cytogenetic analysis is performed based on DGV, inheritance mode, recurrency, and gene contents. For balanced SV, only variations disrupting a known ID gene are retained for interpretation. Small SNV/indel are analyzed following mendelian modes of transmission (*de novo* AD or X linked variations, transmitted AD variation in case of known incomplete penetrance or suspected parental mosaic, maternally X transmission in male patients, homozygous or compound heterozygous variations (SNV or CNV) in autosomal recessive hypothesis). This broad approach ensures that co-occurring mutations (expected in about 5% of cases) will be identified (Yang et al., 2014).

Variants of interest are then recorded in the e-CRF and discussed during the MDM.

#### Multidisciplinary Meeting

Each MDM includes clinician geneticists from the recruiting center, clinicians in charge of the patients’ follow-up (i.e., neuropediatricians, neurologists, pediatricians, etc.), molecular and chromosomal geneticists (from the reference laboratory and its mirror laboratory). To ensure a reasonable number of cases to be reviewed by each MDM, three independent MDMs are organized in parallel, each of them grouping two laboratories and four clinical centers. Each MDM will thus review about 400 inclusions. In order to ensure consistency in decision-making between MDMs, all positive cases from the three MDMs will be validated in a general DEFIDIAG study review meeting.

Each MDM is organized according to the following format: discussion of the list of variants of interest obtained by the simplex 44GPS analysis; then by GSS analysis (for the 196 randomized patients); and finally, by GST. At each step, additional confirmation analysis that would be required in the course of standard care (Sanger, qPCR, FISH, analysis on mRNA, etc.) is recorded on the MDM report and in the e-CRF, for further medico-economic evaluation. The final conclusion concerning the pathogenicity of variant(s) identified by the different approaches will be reached during the session and recorded in the e-CRF MDM conclusion. If additional confirmation methods are required, the reference laboratory will be in charge of this analysis and the case will subsequently be reviewed in a future MDM. The final results are recorded in a research report communicated to the clinical geneticist who included the index case.

Candidate genes or variations (new genes, or putative variations with no obvious pathogenic effect in known genes) are classified as class 3, of unknown significance until the end of the project. Potential reclassification will be managed using standard care procedures such as splice effect, epigenetic signature, functional studies, and cohorts of patients using international collaboration.

#### GS Results Visit and Subsequent 12 months Follow-Up

When the results become available, and regardless of the results (positive or negative), the clinical geneticist who included the patient (and parents) will inform patient/family about the results of the GS during a dedicated visit. These results will be made available approximately 9 months after inclusion in the study. If the study identifies one or several class 3+, 4, or 5 variation(s), the clinical geneticist will explain the type of associated medical condition, its mode of inheritance and the risk of recurrence for a future pregnancy, as well as the modalities of care. All information (examinations, medical or non-medical treatment, medico-social follow-up, etc.) will be collected by the geneticist, assisted by a CRT. The diary kept by the families will be retrieved and integrated into the e-CRF. A new diary will be given to the family for the subsequent 12 months, and the phone contact by the CRT is planned. If the parents or adult with mild ID agreed to participate in the qualitative study, the sociologist/psychologist will then contact them to check that they all still agree to continue the interviews and organize the second interview.

Twelve months after the GS results are made known to the patient, a final visit at the hospital will be organized (this visit can be replaced by a telephone contact, if necessary) to assess the medical condition, collect any results and retrieve the diary. If the parents agreed to participate in the qualitative study, once again, the sociologist/psychologist will then contact them to check that they all still agree to continue the interviews and organize the third interview.

### Medico-Economic Evaluation

A cost-effectiveness analysis will be conducted over the estimated 9–12 months (maximum) required to perform the GS, interpret the data and return the results to the patient. Efficacy will correspond to the diagnostic yield of each of the three strategies being compared. In order to estimate costs from the perspective of the health service, patient management will be divided into three main periods of healthcare consumption (Figure 2). The costs in the medico-economic evaluation will be direct costs, corresponding to medical procedures carried out during period 2. They will include: 1) the costs of consultation with the clinical geneticist in the recruitment centers, 2) the costs of exams preceding the genetic analysis and inclusion in the DEFIDIAG project, and the cost associated with the first blood sample and its transport, 3) the costs associated with any new blood draws required; 4) the costs of genetic analyses, and 5) the costs of any additional and confirmatory tests. Most of these procedures will be valued using social security prices, except for GSS and GST, which will be valued using a micro-costing method (Drummond et al., 2005).

### Impact Studies

Two impact studies will be conducted: the cost of the diagnostic odyssey will first be estimated. It will include the cost of all diagnostic procedures from the first genetics consultation, to inclusion in the DEFIDIAG project (period 1 of Figure 1). The impact of genomic analyses on follow-up will also be assessed. It will be based on a before-after study (period 3 compared to period 1) and will include treatment and diet as well as rehabilitation, psychological and medico-social follow-up.

### Data Management and Data Analyses

#### Data Management

Clinical and paraclinical data as well as the results of the genetic analyses carried out will be entered directly into the dedicated e-CRF by the investigators, helped by CRTs and by biologists and bioinformatics specialists in charge of the GS analyses. The patient diary specific to microcosting and patient follow-up are in paper or electronic format (forms independent of the e-CRF). Each patient is identified by a unique code including: the number of the recruiting center, the inclusion rank, the initials of the patient (first letter of surname and first letter of first name) and a code corresponding to his/her sex. The use of the CleanWEB software makes it possible to carry out checks for missing and incoherent data, and to generate queries immediately after data entry. Requests for corrections may also be generated by the CIC-EC1432 and sent to the recruiting center and/or the reference laboratory. The corrections will be made directly in the e-CRF by the investigators and/or the biologists, assisted by the CRTs. Histories of changes are systematically recorded. A data management plan, specific to the study, integrating centralized monitoring (enabling, for example, comparative monitoring of the distribution of subpopulations between centers and indicators of the quality of sample processing) was prepared before initiating the study in the participating centers.

#### Statistical Analyses

The percentage of ID causal diagnosis identified will be compared between strategies (GST vs reference strategy) using a McNemar test in the overall first-investigation population, then in the seven subgroups of interest. To account for multiple testing, the unilateral alpha risk is set at 0.00278, and also for the secondary comparisons of the diagnostic yield of GST vs. GSS, which will be performed in the dedicated randomized subgroup.

McNemar tests (unilateral alpha risk set at 0.025) will also be used to compare the percentage of causal diagnoses identified by GS and reference strategies in the sub-groups of the individuals coming for a first genetic referral without major non-cerebral abnormality or without epilepsy. This test will also be used in the overall population (first-investigation patients and previously investigated patients) to compare the percentage of causal diagnoses identified by GS strategies in patients with negative CMA. The percentage of causal structural changes identified by GST vs the reference minimal strategy will also be compared using McNemar tests in the first-investigation population (firstly considered all together, and then stratified by subgroups). The number and type of consistent and divergent variants identified with CMA and GS strategies will also be described. The frequency and characteristics of the situations where the causal diagnosis is identified by the reference strategy but not by the GS analyses will be described, as well as the frequency and characteristics of situations where the causal diagnostic is made by GSS and not by GST.

#### Cost-Effectiveness Study

The cost-effectiveness analysis will be performed in the population of randomized patients coming for a first genetic referral and for whom both GS strategies are performed in addition to the reference minimal strategy (i.e., 196 patients). The analysis will be based on the estimation of incremental cost-effectiveness ratios expressed in terms of cost per additional positive diagnosis. Deterministic analysis will take account of progress in technology. In order to manage the uncertainty associated with sampling, a non-parametric bootstrap analysis will be performed.

#### Impact Studies

The costs of finding a diagnosis will be described only in the population of patients who had previously had genetic investigations (50% of the 1,275 index cases), as the mean and standard deviation, if normally distributed, or as median and interquartile range otherwise.

The analysis of the impact of genomic analysis on medical, medico-social, rehabilitation and psychological follow-up after the results are made known, will be performed separately in the previously-explored population (50% of the 1,275 index cases) on the one hand, and in the population attending for a first genetics investigation on the other hand (never-explored patients). Frequencies of follow-up changes between the period prior to inclusion and the period following the results will be calculated with associated 95% confidence intervals. A global analysis will then be performed, regardless of the results of GST. Sub-analyses will be conducted according to the result of GST: positive, negative or uncertain.

#### Qualitative Study

The analysis of the interviews will be based on the following steps: 1) open coding of transcribed interviews, to identify as many topics as possible in the initial corpus; 2) categorization of codes; careful re-reading of the corpus as a whole will be performed to clearly define each category; 3) linking categories and writing of detailed memos and designing explanatory diagrams; 4) integration of the previous steps to identify the key points of the phenomenon; 5) theorization: meticulous and exhaustive construction of the “multidimensionality” and “multicausality” of the phenomenon of the relationships between needs, expectations and hopes, suffering, and the result of genetic analysis. For the psychological aspects, the interviews will be analyzed using the general inductive method (Thomas, 2006), which encompasses the first 3 steps mentioned above.

## Ethics and Dissemination

The study sponsor is the Institut National de la Santé Et de la Recherche Médicale (INSERM). DEFIDIAG study was supported by The French Ministry of Health in the framework of French initiative for genomic medicine (AVIESAN, 2016). An independent international scientific advisory board was constituted in order to make recommendations about the protocol and to evaluate and oversee the scientific and ethical integrity of the study. It is also tasked with evaluating potential sub-study proposals. The Ethics Committee Sud Méditerranée I approved the protocol in June 2019 (under the number 1955/19.05.29.60442). Authorization for detaining nominative databases was granted in March 2020 by the French data privacy commission (Commission Nationale de l’Informatique et des Libertés, CNIL, reference number: 919361). The protocol was registered with ClinicalTrials.gov under the identifier NCT04154891 in November 2019. The first patient was included in March 2020 and the study is expected to be completed by 2023.

We anticipate that the DEFIDIAG study will demonstrate an increase in performance of genetic testing performance in patients (children and adults) affected with ID of unknown etiology. This study will benefit the patient and the family, because it will identify a diagnosis, in turn providing the family with an explanation for the clinical condition, which will at last have a name (heralding the end of their diagnostic odyssey). Finding a diagnosis will enable initiation of appropriate medical therapy and ad hoc care if available, and help in the organization of follow up for the patient, prevent unnecessary medical biological and imaging investigations, authorize reproductive counseling for patient and/or family (prenatal diagnosis, preimplantation diagnosis), enable referral to ad hoc patient and support groups, and contribute to research protocols. Indeed, future perspectives include numerous research projects through the data collected concerning genotype-phenotype analyses, biological integrative analysis of pathways involved in brain development and functioning and last but not least, may help in the elaboration of targeted therapies.

In addition, it is expected that the cost-effectiveness and impact studies will show the efficiency of GST and also the cost-saving and the change in medical practice that can be expected from its implementation. To the best of our knowledge, no data has been published in France on the economic and medical impact of GS compared to the reference strategy or between trio and solo strategies. These arguments are essential to support the decision to implement the appropriate first line GS strategy in diagnostic routine practice, to help public health authorities to determine an adequate tariff with regard to the complete cost of GS, and also to demonstrate the impact of GS on improving patient care. Specifically, we believe it is important to determine the efficiency of a solo strategy, which is less costly in terms of sequencing, but also more pragmatic in many situations where biological relatives are not readily available. However, it is potentially more time consuming to interpret than trio sequencing data. Comparing these two strategies from a medico-economic point of view therefore seems important. We made some important choices concerning the methodology of data collection. The completion of the DEFIDIAG study should enable the key stakeholders to decide on the implementation of GS in France as the first-line test in the care of patients with ID. It will help us to confirm the robustness of the results obtained with traditional data collection by providing GS diagnostic yield estimates in conditions close to routine.

## Datasharing

All requests for the study’s data will be considered by the Defidiag trial steering committee. After the end of the study; and for participants who provide consent, data (excluding data corresponding to the image capture) will be transmitted to and stored at the “CAD” (Collecteur Analyseur de Données) of the French initiative for genomic medicine (AVIESAN, 2016), for potential re-use by other researchers including those not involved in the present study. The conditions for CAD data sharing are being implemented.

## Trial Status

Recruitment is ongoing (955 patients included as of 09/06/2021).

## Full List of Co-Investigators of the DEFIDIAG Study Group:

Meyer Vincent, Centre National de Recherche en Génomique Humaine; Bonneau Dominique, Barth Magalie, Tessarech Marine, Ziegler Alban, CHU d’Angers; Goizet Cyril, Lacombe Didier, Legendre Marine, Margot Henri, Michaud Vincent, Naudion Sophie, Rooryck Thambo Caroline, CHU de Bordeaux; Bournez Marie, Bruel Ange-Line, Colin Estelle, Delanne Julian, Denomme-Pichon Anne-Sophie, Garde Aurore, Moutton Sébastien, Nambot Sophie, Philippe Christophe, Safraou Hana, Sorlin Arthur, Thauvin Christel, Tran-Mau-Them Fréderic, Vitobello Antonio, CHU Dijon-Bourgogne and Inserm UMR1231- Equipe GAD;Dietrich Klaus, Durand Chantal, Marey Isabelle, N’guyen-Morel Marie-Ange, Thevenon Julien, CHU Grenoble-Alpes; Boute Odile, Caumes Roseline, Colson Cindy, Dieux Anne, Ghoumid Jamal, Marsili Luisa, Petit Florence, Vanlerberghe Clémence, Vincent-Delorme Catherine, CHU de Lille; Armand Thibaud, Chatron Nicolas, Curie Aurore, Des Portes Vincent, Edery Patrick, Haye Damien, Labalme Audrey, Lesca Gaëtan, Monin Pauline, Pons Linda, Putoux Audrey, Rossi Massimiliano, Rougeot Christelle, Till Marianne, Hospices Civils de Lyon; Blanchet Patricia, Coubes Christine, Deiller Caroline, Genevieve David, Pinson Lucile, Wells Constance, Willems Marjolaine, CHU de Montpellier; Isidor Bertrand, Mercier Sandra, Nizon Mathilde, Vincent Marie, CHU de Nantes; Amiel Jeanne, Barcia Giulia, Baujat Geneviève, Cormier Valérie, Guimier Anne, Hadj Abdallah Hamza, Malan Valérie, Marlin Sandrine, Marzin Pauline, Michot Caroline, Ormieres Clothilde, Rio Marlène, Romana Serge, Hôpital Necker-Enfants Malades (AP-HP); Afenjar Alexandra, Burglen Lydie, Charles Perrine, Courtin Thomas, Heide Solveig, Keren Boris, Lehalle Daphné, Mignot Cyril, Mouthon Linda, Whalen Sandra, Groupe Hospitalier Pitié-Salpêtrière (AP-HP); Fradin Mélanie, Jean-Marcais Nolwenn, Lavillaureix Alinoë, Morel Godelieve, Pasquier Laurent, Quelin Chloé, Riou Audrey, Ugolin Mélissa, CHU de Rennes; Brehin Anne-Claire, Cassinari Kévin, Chambon Pascal, Goldenberg Alice, Guerrot Anne-Marie, Joly-Helas Géraldine, Lecoquierre François, Lemeur Nathalie, Nicolas Gaël, Saugier-Veber Pascale, Vera Gabriella, CHU de Rouen; El Chehadeh Salima, Calmels Nadège, Haushalter Virginie, Maillard Pierre-Yves, Muller Jean, Philippe Anaïs, Piton Amélie, Schaefer Elise, Scheidecker Sophie, Schluth-Bolard Caroline, Hôpitaux Universitaires de Strasbourg; Busa Tiffany, Philip-Sarles Nicole, Riccardi Florence, Sigaudy Sabine, Hôpital de la Timone (Hôpitaux Universitaires de Marseille); Nitschke Patrick, Institut Imagine.
