# Supplementary material for: Genome Sequencing for Genetics Diagnosis of Patients With Intellectual Disability: The DEFIDIAG Study
Source: Front Genet. 2022 Feb 1;12:766964. doi: 10.3389/fgene.2021.766964 (PMC8845475; doi:10.3389/fgene.2021.766964)
Supplement: Supplementary file 1 [file Table1.DOCX]

Supplementary Material

# Supplementary Table S1. Genes included in the 44 ID Panel

| \| **Gene** \| **Mean diagnostic yield according to literature** \| **size (kb)** \| **transmission** \| **NM_** \| **NP_** \| \| --- \| --- \| --- \| --- \| --- \| --- \| \| **ANKRD11** \| 0.53% \| 8 034 \| AD \| NM_001256182 \| NP_001243111 \| \| **ARID1B** \| 0.74% \| 7 376 \| AD \| NM_020732 \| NP_065783 \| \| **ATRX** \| 0.21% \| 7 559 \| XL \| NM_000489 \| NP_000480 \| \| **CASK** \| 0.27% \| 2 835 \| XL \| NM_003688 \| NP_003679 \| \| **CTNNB1** \| 0.37% \| 2 346 \| AD \| NM_001904 \| NP_001895 \| \| **CUL4B** \| 0.10% \| 2 805 \| XL \| NM_003588 \| NP_003579 \| \| **DLG3** \| 0.05% \| 2 784 \| XL \| NM_021120 \| NP_066943 \| \| **DYRK1A** \| 0.58% \| 2 417 \| AD \| NM_001396 \| NP_001387 \| \| **EP300** \| 0.16% \| 7 245 \| AD \| NM_001429 \| NP_001420 \| \| **FOXP1** \| 0.16% \| 2 615 \| AD \| NM_032682 \| NP_116071 \| \| **GATAD2B** \| 0.10% \| 1 782 \| AD \| NM_020699 \| NP_065750 \| \| **GRIA3** \| 0.05% \| 3 248 \| XL \| NM_000828 \| NP_000819 \| \| **GRIN1** \| 0.16% \| 3 090 \| AD \| NM_007327 \| NP_015566 \| \| **GRIN2A** \| 0.10% \| 1 395 \| AD \| NM_001134407 \| NP_001127879 \| \| **GRIN2B** \| 0.16% \| 4 455 \| AD \| NM_000834 \| NP_000825 \| \| **IL1RAPL1** \| 0.10% \| 2 091 \| XL \| NM_014271 \| NP_055086 \| \| **IQSEC2** \| 0.16% \| 4 689 \| XL \| NM_001111125 \| NP_001104595 \| \| **KDM5C** \| 0.16% \| 4 924 \| XL \| NM_004187 \| NP_004178 \| \| **KMT2A (MLL)** \| 0.27% \| 12 066 \| AD \| NM_001197104 \| NP_001184033 \| \| **MECP2** \| 0.47% \| 1 612 \| XL \| NM_001110792 \| NP_001104262 \| \| **MED13L** \| 0.21% \| 6 633 \| AD \| NM_015335 \| NP_056150 \| \| **NAA10** \| 0.10% \| 792 \| XL \| NM_003491 \| NP_003482 \| \| **PQBP1** \| 0.11% \| 856 \| XL \| NM_005710 \| NP_005701 \| \| **PTCHD1** \| 0.16% \| 2 667 \| XL \| NM_173495 \| NP_775766 \| \| **SATB2** \| 0.63% \| 2 237 \| AD \| NM_001172509 \| NP_001165980 \| \| **SCN2A** \| 0.84% \| 6 110 \| AD \| NM_021007 \| NP_066287 \| \| **SCN8A** \| 0.16% \| 6 082 \| AD \| NM_014191 \| NP_055006 \| \| **SETBP1** \| 0.16% \| 4 980 \| AD \| NM_015559 \| NP_056374 \| \| **SHANK3** \| 0.26% \| 5 302 \| AD \| NM_001080420 \|  \| \| **SLC16A2** \| 0.10% \| 1 842 \| XL \| NM_006517 \| NP_006508 \| \| **SLC2A1** \| 0.16% \| 1 941 \| AD \| NM_006516 \| NP_006507 \| \| **SLC6A8** \| 0.10% \| 3 075 \| XL \| NM_005629 \| NP_005620 \| \| **SLC9A6** \| 0.16% \| 2 106 \| XL \| NM_001042537 \| NP_001036002 \| \| **SMARCA2** \| 0.21% \| 4 892 \| AD \| NM_003070 \| NP_003061 \| \| **SMC1A** \| 0.16% \| 3 883 \| XL \| NM_006306 \| NP_006297 \| \| **STXBP1** \| 0.74% \| 1 895 \| AD \| NM_003165 \| NP_003156 \| \| **SYNGAP1** \| 0.63% \| 4 178 \| AD \| NM_006772 \| NP_006763 \| \| **TBR1** \| 0.26% \| 2 049 \| AD \| NM_006593 \| NP_006584 \| \| **TCF4** \| 0.47% \| 2 657 \| AD \| NM_001243226 \| NP_001230155 \| \| **UPF3B** \| 0.05% \| 1 452 \| XL \| NM_080632 \| NP_542199 \| \| **FOXG1** \| 0.10% \| 1 470 \| AD \| NM_005249 \| NP_005240 \| \| **CDKL5** \| 0.11% \| 3 093 \| XL \| NM_003159 \| NP_003150 \| \| **RAI1** \| 0.10% \| 6 245 \| AD \| NM_030665 \| NP_109590 \| \| **WDR45** \| 0.32% \| 1 550 \| XL \| NM_007075 \| NP_009006 \| \| **44 genes** \| **11.20%** \| **163 355** \|  \|  \|  \| |  |  |  |  |
| --- | --- | --- | --- | --- | --- | --- | --- | --- | --- | --- | --- | --- | --- | --- | --- | --- | --- | --- | --- | --- | --- | --- | --- | --- | --- | --- | --- | --- | --- | --- | --- | --- | --- | --- | --- | --- | --- | --- | --- | --- | --- | --- | --- | --- | --- | --- | --- | --- | --- | --- | --- | --- | --- | --- | --- | --- | --- | --- | --- | --- | --- | --- | --- | --- | --- | --- | --- | --- | --- | --- | --- | --- | --- | --- | --- | --- | --- | --- | --- | --- | --- | --- | --- | --- | --- | --- | --- | --- | --- | --- | --- | --- | --- | --- | --- | --- | --- | --- | --- | --- | --- | --- | --- | --- | --- | --- | --- | --- | --- | --- | --- | --- | --- | --- | --- | --- | --- | --- | --- | --- | --- | --- | --- | --- | --- | --- | --- | --- | --- | --- | --- | --- | --- | --- | --- | --- | --- | --- | --- | --- | --- | --- | --- | --- | --- | --- | --- | --- | --- | --- | --- | --- | --- | --- | --- | --- | --- | --- | --- | --- | --- | --- | --- | --- | --- | --- | --- | --- | --- | --- | --- | --- | --- | --- | --- | --- | --- | --- | --- | --- | --- | --- | --- | --- | --- | --- | --- | --- | --- | --- | --- | --- | --- | --- | --- | --- | --- | --- | --- | --- | --- | --- | --- | --- | --- | --- | --- | --- | --- | --- | --- | --- | --- | --- | --- | --- | --- | --- | --- | --- | --- | --- | --- | --- | --- | --- | --- | --- | --- | --- | --- | --- | --- | --- | --- | --- | --- | --- | --- | --- | --- | --- | --- | --- | --- | --- | --- | --- | --- | --- | --- | --- | --- | --- | --- | --- | --- | --- | --- | --- | --- | --- | --- | --- | --- | --- | --- | --- | --- | --- | --- | --- | --- | --- | --- | --- | --- | --- | --- | --- |

# Supplementary Table S2: Tasks of reference and mirror laboratories (DEFIDIAG study)

| **Action as reference laboratory** | **Actions as mirror laboratory** |
| --- | --- |
| - Pre-analytical processing of trio samples: checking for sample conformity as well as clinical data and consent recording  - DNA extraction for the trio  - Quality checks after extraction  - Batch transmission to CNRGH |  |
| Genome sequencing at the CNRGH | |
| - Verification of the quality control report  - Verification of the trio concordance |  |
| - Analysis of GS trio on the data of the GS according to the protocol defined  - Develop the list of suspected causal variants, at the end of the trio analysis and collect them in the e-CRF with any potential corresponding confirmatory analyses to be performed, to be discussed in the MDM | - Analysis of the simplex data for the standard 44 genes extracted from the GS data (exonic ± 20 intronic bases).  - Analysis of simplex data on the data of the GS according to the protocol defined for 196 randomized patients  - Develop the list of suspected causal variants, at the end of the simplex analysis and collect them in the e-CRF as well as the potential corresponding confirmatory analyses to perform, to be discussed in the MDM |
| Present and discuss the identified variants during the MDM.  The MDM will begin with the presentation of the variants identified by the simplex analysis and then the presentation of the variants identified by the trio analysis to ensure optimal blindness. A synthesis will be performed at the end of the meeting with the two results, simplex and trio.  The biological analyses will be validated during the MDM and the conclusion will be recorded in the eCRF with the following situations:   1. Etiological diagnosis confirmed: diagnosis of ID can be made, 2. Uncertain etiological diagnosis: one or more highly suspected genetic abnormalities have been found but are of unknown clinical significance. 3. Absence of diagnosis   During the same MDM, the following elements are also discussed   - Confirmation studies needed (mRNA analysis, Sanger studies in relatives, metabolic studies, etc*.*) - Selection of cases that will be proposed for inclusion in the biobank project. | |
| - Carry out any confirmatory analyses decided at the MDM  - Resubmit the file to a future MDM when necessary, after the results of the confirmatory analyses |  |
| - Produce a research report based on the MDM's decision |  |
